# A study on identity conflict, self-concept clarity, and dual career adaptability among collegiate athletes

**DOI:** 10.3389/fspor.2026.1877707

**Published:** 2026-09-14

**Authors:** Hsueh-Chung Wang, Wan Chen Lu, Hsin Chuan Chen

**Affiliations:** 1 Office of Physical Education, Ming Chi University of Technology, New Taipei, Taiwan; 2 Department of Athletics, National Taiwan University, Taipei, Taiwan; 3 Bachelor Program of International Sport Affairs, National Taiwan University, Taipei, Taiwan

**Keywords:** career transition, conservation of resources theory (COR), personal resources, role strain, self-regulation

## Abstract

The study examined the prospective associations among academic–athletic identity conflict (role interference), self-concept clarity, and dual career adaptability among Taiwanese collegiate student-athletes. Drawing on Conservation of Resources theory, self-concept clarity was conceptualised as a potentially relevant personal psychological resource associated with student-athletes’ adaptation to concurrent academic and athletic demands. A two-wave prospective association design with a cross-sectional first stage (four-month interval) was employed. Identity conflict and self-concept clarity were assessed at Time 1, and dual career adaptability was assessed at Time 2. Valid paired responses were obtained from 414 student-athletes. A statistically significant indirect prospective association was observed through self-concept clarity: Time 1 identity conflict was negatively associated with Time 1 self-concept clarity, which was subsequently associated with lower Time 2 dual career adaptability measured 4 months later. The bootstrap 95% confidence interval for the indirect association did not include zero. Because baseline adaptability was not controlled for at Time 1, these findings represent prospective cross-lagged level-associations rather than intra-individual changes or development in adaptability over time. The findings are consistent with Conservation of Resources theory and suggest that self-concept clarity may represent one of several psychological resources relevant to dual career adaptation within the Taiwanese socio-cultural context.

## 1 Introduction

In recent years, the development and adaptation of student-athletes’ dual career (DC) have become core issues of high interest within the international sport psychology community. A dual career refers to athletes’ pursuit of athletic excellence while simultaneously managing their educational or vocational development (1). However, the implementation of a dual career is deeply influenced by the socio-cultural contexts and systems of different countries (2). Specifically in Taiwan, the scarcity of professional sporting pathways means that higher education frequently marks the final chapter of an athlete’s competitive journey, compelling them to confront an inevitable career transition immediately after graduation (3). Furthermore, the Asian socio-ecological system places high expectations on academic achievement and future stable employment (4). Consequently, Taiwanese collegiate student-athletes face immense pressure to simultaneously satisfy “high-intensity athletic training” and “strict academic demands” within a limited timeframe.

From the perspective of Conservation of Resources (COR) theory (5), the high demands and time pressures associated with dual roles place student-athletes in a high-stress environment that may be associated with resource strain. COR theory is particularly appropriate for understanding this process because it conceptualizes stress as arising not only from demanding circumstances themselves, but also from the threat of resource loss, actual resource loss, or insufficient returns following substantial resource investment (6). Student-athletes must continuously invest finite resources, including time, physical energy, attention, and emotional capacity, in both academic and athletic domains. When the demands of these domains are incompatible, investment in one role may reduce the resources available for fulfilling the other, thereby transforming academic–athletic identity conflict into a process of cumulative resource depletion. When student-athletes attempt to strike a balance between two highly demanding social roles—student and athlete—they often experience identity conflict. Identity conflict refers to the contradictions and interference that arise when different social roles compete for an individual’s limited time, energy, and psychological resources (7). Previous applications of COR theory to inter-role conflict have similarly demonstrated that persistent demands across competing life domains can drain resources and subsequently produce psychological strain (8). More specifically, Wendling, E., Kellison, T. B., & Sagas, M (9). proposed COR theory as a suitable framework for explaining how academic–athletic role conflict affects collegiate student-athletes because it simultaneously accounts for resource depletion and the protective functions of personal and social resources. Thus, COR theory enables the present study to move beyond examining whether identity conflict impairs dual career adaptability and to explain the resource-based psychological process through which this impairment occurs. While previous literature has predominantly focused on the direct negative impact of identity conflict on athletes’ adaptation, few studies have unpacked this psychological status to determine how student-athletes’ resources become depleted and which resources may buffer this process. To manage the various challenges of a dual career, athletes must possess adequate dual career adaptability (10). Clarifying the internal psychological mechanisms linking identity conflict and adaptability therefore represents an important gap in current cross-cultural dual career research.

Within the COR theory, the study conceptualises self-concept clarity as a potentially relevant personal resource for student-athletes managing identity conflict. Self-concept clarity refers to the extent to which individuals’ beliefs about themselves are clearly defined, internally consistent, and stable over time (11). Stress may arise when valued resources are threatened or when individuals must repeatedly invest resources in managing persistent demands (6). Accordingly, the incompatible expectations associated with student and athlete roles may require sustained cognitive and emotional effort. Related research has shown that self-concept clarity statistically mediates the association between perceived stress and subjective well-being, with greater stress being associated with lower self-concept clarity and, in turn, poorer well-being (12). These findings provide a basis for proposing that persistent academic–athletic identity conflict may be associated with reduced clarity and coherence in student-athletes’ self-perceptions.

Self-concept clarity may also be relevant to dual career adaptability because career adaptation requires individuals to identify personally meaningful goals and organize their responses to career-related demands and transitions (13). Uğurlar and Wulff (14) found that individuals with higher self-concept clarity were better able to identify goal-relevant information and performed more accurately in social decision-making tasks. Similarly, Bechtoldt, De Dreu, C. K. W., Nijstad, and Zapf (15) reported that higher self-concept clarity was associated with more active and cooperative responses to interpersonal conflict, partly through lower levels of rumination. Although these findings were not obtained from student-athlete populations, they suggest that a clear and coherent self-concept may support goal-directed decision-making and constructive responses to conflicting demands. Therefore, the present study proposes that self-concept clarity may partially account for the relationship between identity conflict and dual career adaptability.

In summary, this study aims to apply COR theory to explore whether self-concept clarity (a critical psychological resource) exhibits a prospective indirect relationship between identity conflict (a resource-depleting stressor) and dual career adaptability among Taiwanese collegiate student-athletes. This study proposes the following refined hypotheses:

H1: Time 1 student-athletes’ identity conflict will be significantly and negatively associated with their Time 1 self-concept clarity.
H2: Time 1 self-concept clarity will be significantly and positively associated with Time 2 dual career adaptability measured four months later.
H3: Time 1 identity conflict will exhibit a statistically significant indirect prospective association with Time 2 dual career adaptability through Time 1 self-concept clarity.

## 2 Method

### 2.1 Participants and procedure

The study employed a two-wave design to investigate the mediating role of self-concept clarity between identity conflict and dual career adaptability among Taiwanese collegiate student-athletes. The four-month interval was chosen to ensure that student-athletes had experienced a full academic semester.

During the first wave (T1) at the beginning of the semester, student-athletes were facing academic planning for the new semester alongside the start of a new training cycle. Participants were recruited voluntarily after the research team contacted the coaches of collegiate athletic teams and requested their assistance in distributing information about the study. All 494 student-athletes who agreed to participate provided complete T1 questionnaires. This timing was intended to capture their initial experiences of identity conflict when confronted with dual role demands, as well as their concurrent self-concept clarity. The second wave (T2) was conducted four months later at the end of the semester, providing a prospective assessment of dual career adaptability at the end of the academic semester. Dual career adaptability was intentionally measured at T2 to examine its association with identity conflict and self-concept clarity measured at T1.

Prior to data collection, ethical approval was obtained from the National Taiwan University Research Ethics Committee (Approval No. 202312HS009). Participants were explicitly informed of the voluntary and confidential nature of the study. No incentives were provided for participation. De-identified data were stored in password-protected files accessible only to the research team. Informed consent was obtained from all participants prior to participation. At T1, a total of 494 student-athletes voluntarily participated in the study and completed the questionnaire in full. These participants were subsequently invited to complete the T2 questionnaire. Some participants did not participate in T2 because of graduations, academic or training commitments, while some T2 questionnaires were excluded because they were incomplete or could not be validly matched with the corresponding T1 responses. At both assessment waves, participants were instructed to generate a unique, de-identified matching code (comprising the last three digits of their personal mobile number combined with their birth month and day). This procedural control allowed the research team to link the Time 1 and Time 2 responses longitudinally without collecting any direct personal identifiers (such as names or student IDs). After matching the T1 and T2 responses using these de-identified codes and removing invalid or incomplete questionnaires, 414 valid paired responses were retained for the final analysis, representing a paired-response retention rate of 83.8%.

Among the 414 participants, 56.0% were male. Their mean age was 20.24 years. Taekwondo (23.9%), volleyball (22.7%), and table tennis (18.8%) were the three largest sports specialities among the student-athletes. The sample also included student-athletes participating in baseball, softball, basketball, and tennis. The mean sport-specific training experience was 10.52 years. The participants represented 19 sports and 2 competitive levels across 10 universities, covering a large portion of the student-athlete population in Taiwan. However, because the study focused on the academic–athletic role demands commonly experienced by collegiate student-athletes, the sample was analysed as an overall student-athlete population rather than being divided into sport-, institution-, or competition-level subgroups.

### 2.2 Measurement

#### 2.2.1 Identity conflict

This study adopted the 12-item Student-Athlete Role Interference Scale developed by Settles, Sellers and Damas (16). The Chinese version of this scale, translated by Chang (17), aims to assess the degree to which participants perceive their athletic and student roles as interfering with each other. A sample item is ’Some student-athletes feel that sports limit their academic performance.’ Participants responded using a 7-point Likert scale ranging from 1 (*not really true of me*) to 7 (*really true of me*), with higher average scores indicating more severe identity conflict. In the study, identity conflict was operationalised as perceived academic–athletic role interference, a central, contextually observable manifestation of tension between student and athlete identities. Conflicting demands related to time, performance, and role expectations constitute important ways in which student-athletes experience identity conflict in their everyday dual career contexts. Accordingly, the Student-Athlete Role Interference Scale provides an appropriate measure of the practical expression of identity conflict examined in this study. Although identity conflict may also involve broader psychological tensions between self-definitions, the present operationalisation intentionally focuses on the role-based interference most directly relevant to student-athletes’ efforts to balance sport and education. The internal consistency (Cronbach’s α) of the scale in the present sample was.872.

#### 2.2.2 Self-concept clarity

This study utilized the 12-item Self-Concept Clarity Scale developed by Campbell, Trapnell, Heine, Katz, Lavallee and Lehman (11). The Chinese version of this scale, translated by Chiu (18), assesses the perceived certainty, temporal stability, and internal consistency of an individual’s self-beliefs. A sample item is ‘My beliefs about myself often conflict with one another.’ Participants rated each item on a 6-point Likert scale (6 = *strongly agree*; 1 = *strongly disagree*). All items were reverse-coded prior to computing the total score, with higher average scores indicating higher self-concept clarity. In the present sample, the scale demonstrated excellent internal consistency (Cronbach’s α = 0.947).

#### 2.2.3 Dual career adaptability

Dual career adaptability was assessed using the Career Adapt-Abilities Scale–Dual Career Form (CAAS-DC) (10), using the Chinese version translated by Hu, Lin, Hu and Lu (19). The 27-item instrument evaluates five dimensions: concern, dual career concern, control, curiosity, and confidence. A sample item is “Thinking about what my future will be like.” Participants rated items on a 5-point Likert scale ranging from 1 (not a strength) to 5 (greatest strength), with higher mean scores indicating greater dual career adaptability. In the current sample, the overall scale exhibited high internal consistency (Cronbach’s α = 0.958).

#### 2.2.4 Control variables

Following Steele, Van Rens, and Ashley (20), gender was controlled as a dummy variable (0 = male, 1 = female) because male and female student-athletes may differ in academic and athletic role identification. Additionally, based on Miller and Kerr (21), age and years of sport-specific training were included as control variables to account for developmental shifts in focus toward academic and post-athletic career considerations across educational stages. Therefore, this study used student-athletes’ gender, age and years of training as control variables.

#### 2.2.5 Data analysis, regression diagnostics, and common method variance

Statistical assumptions were rigorously evaluated on model regression residuals rather than raw manifest variables (22). In SPSS, residual normality was verified using standardized residual histograms and Normal P-P plots. Multicollinearity was ruled out via Coefficients and Collinearity Diagnostics tables (VIF<2.50, Tolerance>.10). Linearity and homoscedasticity were supported by standardized residual scatterplots showing a uniform rectangular error dispersion around zero. Multivariate outliers and influential cases were checked via Residuals Statistics (Cook’s distance, leverage, and Mahalanobis distance). Finally, Independent-Samples T-Tests confirmed no statistically significant baseline differences between dropouts (*n* = 80) and retained participants (*N* = 414) across all Time 1 measures—specifically identity conflict, self-concept clarity, gender, age, and years of training (*p* > 0.05).

Primary prospective path analysis and indirect associations were evaluated using Model 4 of the PROCESS macro for SPSS (23) with 5,000 bias-corrected bootstrap resamples and 95% confidence intervals.

Because all primary variables were assessed through self-report questionnaires, both procedural remedies and statistical diagnostic assessments were employed to mitigate and evaluate potential common method bias (24, 25). Procedurally, three strategies were implemented during research design and administration: (1) Temporal Separation: A four-month time lag was introduced between the collection of Time 1 measures (Identity Conflict and Self-Concept Clarity) and Time 2 measures (Dual Career Adaptability), interrupting the cognitive continuity of response contexts; (2) Psychometric Separation and Format Heterogeneity: Scale items were physically separated across distinct section pages, utilizing varied Likert response formats (7-point scale for Identity Conflict, 6-point scale for Self-Concept Clarity, and 5-point scale for Dual Career Adaptability) and incorporating reverse-coded items (SCC scale) to reduce pattern-set responding; and (3) Anonymity and Reduced Evaluative Threat: Participants were explicitly assured of electronic response confidentiality and informed that there were no right or wrong answers, thereby minimizing social desirability bias and evaluation apprehension. Statistically, Harman’s single-factor test revealed that the first unrotated factor accounted for 33.38% of the total variance (well below the 50% threshold), confirming that common method bias does not pose a serious threat to the validity of the findings.

#### 2.2.6 Confirmatory factor analysis

Confirmatory factor analyses (CFAs) using Maximum Likelihood (ML) estimation in LISREL 8.80 were conducted on measures to evaluate construct validity and measurement model fit prior to structural path testing. Complete item-level parameter estimates—including unstandardized loadings (B), standard errors (SE), t-values, completely standardized factor loadings (*λ*), residual variances (*δ*), item R² values, Composite Reliability (CR), and Average Variance Extracted (AVE)—are fully documented in Supplementary Tables A1.1–A1.4.

For the Student-Athlete Identity Conflict Scale (12 items), three pairs of error covariances (RI8–RI9, RI9–RI11, and RI10–RI11) were specified based on substantive item-content semantic redundancy and method variance (22, 26, 27). Specifically, RI8 (‘coach lack of academic support’) and RI9 (’sport specialization limiting academic performance’) share specific situational uniqueness regarding structural athletic barriers to education. RI9 and RI11 (’stress from sacrificing academics for sport specialization’) share specific operational overlap regarding academic sacrifices driven by athletic specialization. Similarly, RI10 (‘peer evaluation anxiety regarding college admission’) and RI11 share specific affective variance pertaining to subjective psychological strain and social evaluation anxiety. Modelling these error covariances accommodates shared item-pair uniqueness without confounding the underlying unidimensional construct (CR = 0.94, AVE = 0.59). The overall model fit indices were: *χ*²(51) = 361.56, *p* < 0.001 (*χ*²/df = 7.09), RMSEA = 0.120 [90% CI (.110,.130)], SRMR = 0.069, CFI = 0.930, NFI = 0.920, and NNFI = 0.910.

For the Self-Concept Clarity Scale (12 items), all items exhibited statistically significant standardized factor loadings ranging from.69 to.84 (*p* < 0.001), supporting robust unidimensional convergent validity (CR = 0.95, AVE = 0.60) without *post-hoc* residual modifications. The model fit indices were: *χ*²(54) = 535.75, *p* < 0.001 (*χ*²/df = 9.92), RMSEA = 0.180 [90% CI (.170,.190)], SRMR = 0.061, CFI = 0.950, NFI = 0.950, and NNFI = 0.940.

Although point estimates of RMSEA for Identity Conflict (RMSEA = 0.120) and Self-Concept Clarity (RMSEA = 0.180) exceeded traditional thresholds, methodological research indicates that RMSEA suffers from severe upward bias in simple unidimensional models with small degrees of freedom (df) and moderate sample sizes (*N* = 414) (37). Following Hu and Bentler’s (28) combinational evaluation rules, when SRMR ≤ 0.08 (our measures range from.032 to.069) and CFI ≥ 0.95 (ranging from.930 to.990), Type I and Type II error rates are minimized, confirming that the observed covariance structure is adequately reproduced.

For Dual Career Adaptability (27 items), evaluated under a 5-factor model in LISREL, the CFA results yielded good overall model fit indices: *χ*²(314) = 623.19, *p* < 0.001 (*χ*²/df = 1.99), RMSEA = 0.049 [90% CI (.043,.054)], SRMR = 0.032, CFI = 0.990, NFI = 0.980, and NNFI = 0.990. Subscale Composite Reliability values ranged from.88 to.92, and Average Variance Extracted values ranged from.51 to.66, supporting good construct validity.

## 3 Results

### 3.1 Descriptive analyses

This study employed descriptive statistics to present the distribution of student-athletes’ identity conflict, self-concept clarity, dual career adaptability, and the control variables (gender, age, and years of training). As shown in Table 1, the mean score for student-athletes’ identity conflict was 4.00 (*SD* = 0.96), self-concept clarity was 3.22 (*SD* = 0.93), and dual career adaptability was 3.92 (*SD* = 0.59).

**Table 1:** Results of variable distribution and correlation analysis.

Variables	1	2	3	4	5	6
1. gender	–					
2. age	-0.03	–				
3. years of training	-0.20**	.46**	–			
4. identity conflict	.04	-0.01	.09	–		
5. self-concept clarity	.07	.08	.01	-0.35**	–	
6. dual career adaptability	.11*	.07	-0.02	-0.02	.16**	-
Mean	-	20.24	10.52	4.00	3.22	3.92
Standard Deviation	-	1.75	3.20	.96	.93	.59

*N* = 414. Gender was coded as a dummy variable.

* *p* < 0.05.

** *p* < 0.01.

*** *p* < 0.001.

This study utilized Pearson’s correlation analysis to examine the relationships between variables. As shown in Table 1, regarding the control variables, gender showed a significant negative correlation with years of training (*r* = -0.20, *p* < 0.01) and a significant positive correlation with dual career adaptability (*r* = 0.11, *p* < 0.05). Age was significantly positively correlated with years of training (*r* = 0.46, *p* < 0.01).

Regarding the main research variables, student-athletes’ identity conflict showed a significant negative correlation with self-concept clarity (*r* = -0.35, *p* < 0.01); self-concept clarity was significantly positively correlated with dual career adaptability (*r* = 0.16, *p* < 0.01). However, the correlation between identity conflict and dual career adaptability did not reach a significant level (*r* = -0.02, *p* > 0.05).

### 3.2 Path decomposition, suppression analysis, and prospective indirect association

To rigorously evaluate the dynamic relationship between Time 1 Identity Conflict, Time 1 Self-Concept Clarity, and Time 2 Dual Career Adaptability, a comprehensive effect decomposition was performed using Model 4 of the PROCESS macro (23) with specified control variables (gender, age, and training years) and 5,000 bootstrap resamples. The unstandardized and completely standardized parameters—including total, direct, and indirect effects, standard errors, 95% bias-corrected bootstrap confidence intervals, and exact *p*-values—are coherently integrated in Table 2.

**Table 2:** Integrated regression and effect decomposition results for the prospective indirect model (*N* = 414).

Model & Path/Parameter	Unstd. B	*SE*	*t/Boot SE*	*p*-value	95% CI [LL, UL]	Std. *β*
Mediator Model: Self-Concept Clarity(T1), R^2^ = 0.133 F _(4, 409)_ = 15.68, *p* < 0.001						
Constant	3.685	.543	6.785	<.001	[2.618, 4.753]	—
Gender (0 = Male,1= Female)	.167	.088	1.894	.059	[−0.006, 0.341]	.088
Age	.037	.028	1.327	.185	[−0.018, 0.091]	.069
Years of Training	.005	.016	0.292	.770	[−0.026, 0.035]	.015
Identity Conflict (T1)	−.339	.045	−7.558	<.001	[−0.427, −0.251]	−.350
Outcome Model: Dual Career Adaptability (T2), R^2^ = 0.040 F _(5, 408)_ = 3.42, *p* = 0.005						
Constant	2.980	.383	7.790	<.001	[2.228, 3.731]	—
Gender	.107	.059	1.808	.071	[−0.009, 0.223]	.089
Age	.028	.019	1.521	.129	[−0.008, 0.065]	.082
Years of Training	−.008	.010	−0.754	.451	[−0.028, 0.013]	−.042
Identity Conflict (T1) [Direct Effect, c’]	.02	.032	0.627	.531	[−0.043, 0.083]	.032
Self-Concept Clarity (T1) [Mediator, b]	.098	.033	2.973	.003	[0.033, 0.163]	.154
Effect Decomposition Summary (Identity Conflict T1 → Dual Career Adaptability T2) (5,000 Bootstrap)						
Total Effect (c)	−.013	.032	−0.414	.679	[−0.076, 0.050]	−.022
Direct Effect (c’)	.020	.032	0.627	.531	[−0.043, 0.083]	.032
Indirect Effect (a × b) via SCC	−.033	.015	—	.012	[−0.069, −0.007]	−.054

*N* = 414. Gender was dummy-coded (0 = Male, 1 = Female). Bootstrap resamples = 5,000. *For the indirect effect, Boot SE and 95% Bias-Corrected Bootstrap Confidence Intervals are reported. Completely standardized indirect effect β = -0.054 [Boot SE = 0.024, 95% Boot CI (-0.112, -0.011)]. The direct effect (c’ =  + 0.020) and indirect effect (a × b = −0.033) operate in opposite directions, resulting in a near-zero total association (c = −0.013, *p* = 0.679; bivariate r = -0.02), which reflects inconsistent mediation (or statistical suppression) (22, 29, 30).

* *p* < 0.05,.

** *p* < 0.01,.

*** *p* < 0.001.

As presented in Table 2, Time 1 Identity Conflict was significantly and negatively associated with Time 1 Self-Concept Clarity [*B* = −0.339, *SE* = 0.045, *t* = −7.558, *p* < 0.001, 95% CI(−0.427,−0.251)]. In turn, Time 1 Self-Concept Clarity exhibited a positive prospective association with Time 2 Dual Career Adaptability measured four months later [*B* = 0.098, *SE* = 0.033, *t* = 2.973, *p* = 0.003, 95% CI(0.033,0.163)]. The indirect prospective path through Self-Concept Clarity was statistically significant [*B* = −0.033, Boot *SE* = 0.015, 95% Bootstrap CI[−0.069,−0.007]; completely standardized indirect effect *β*=−0.054,95% Bootstrap CI[−0.112,−0.011]].

Notably, the direct association between Identity Conflict and Dual Career Adaptability was slightly positive but non-significant [*B* = 0.020, *SE* = 0.032, *t* = 0.627, *p* = 0.531, 95% CI(−0.043,0.083)]. Because the direct effect (*B* = 0.020) and indirect effect (*B* = −0.033) operate in opposite directions, they result in a near-zero total association [*B* = −0.013, *SE* = 0.032, *t* = −0.414, *p* = 0.679,95% CI(−0.076,0.050)], which aligns with the non-significant bivariate correlation reported in Table 1 (*r* = −.02, *p* > 0.05). In accordance with methodological literature (22, 29, 30), this pattern reflects inconsistent mediation (or statistical suppression) rather than a simple, homogeneous resource-depletion pathway. Specifically, the negative indirect effect through Self-Concept Clarity is suppressed at the bivariate level by the weak positive direct path, cancelling out the overall total relationship.

Because Time 1 baseline adaptability was not measured or controlled for, this statistically significant indirect effect in a two-wave design with a cross-sectional first stage demonstrates a modest prospective level-association rather than explaining intra-individual changes over time or establishing Self-Concept Clarity as an absolute causal mechanism (31, 32).

## 4 Discussions

This study applied Conservation of Resources (COR) theory (5) to examine the prospective associations among identity conflict, self-concept clarity, and dual career adaptability in Taiwanese collegiate student-athletes. The results identified a statistically significant indirect prospective association between Time 1 identity conflict and Time 2 dual career adaptability through Time 1 self-concept clarity. This pattern provides preliminary empirical support for considering self-concept clarity as one possible psychological mechanism linking multiple role pressures to student-athletes’ dual career adjustment. However, the theoretical interpretation of this pathway requires careful qualification based on our research design. First, because identity conflict and self-concept clarity were measured concurrently at Time 1, their relationship is fundamentally cross-sectional, and temporal precedence cannot be established. Reverse directionality remains a plausible alternative explanation: student-athletes with lower self-concept clarity may lack stable internal self-beliefs, rendering them more vulnerable to perceiving heightened interference between their academic and athletic roles (11, 16). Second, because Time 1 baseline dual career adaptability was not controlled for, our analysis does not show that these predictors explain changes, development, or improvement in adaptability over time. It demonstrates only that Time 1 predictors are associated with the level of adaptability measured four months later.

Within the framework of COR theory (5), this pattern is consistent with the possibility that identity conflict, as a chronic stressor arising from competing athletic and academic demands (7), may be related to dual career adaptability through its association with relevant psychological resources rather than through a detectable direct association. Specifically, persistent role strain may be associated with lower temporal stability and internal consistency in individuals’ self-beliefs, thereby contributing to lower self-concept clarity (11). This interpretation is broadly consistent with previous findings linking stress to lower self-concept clarity and, in turn, poorer psychological well-being (12). Because career adaptability involves psychosocial resources that help individuals manage career-related tasks and transitions (13), lower self-concept clarity may make it more difficult for student-athletes to identify personal goals and organize adaptive responses. From the perspective of the Holistic Athletic Career Model (HACM), such difficulties may be relevant when student-athletes simultaneously encounter academic, athletic, and psychosocial transitions (1).

The principal contribution of this study lies in identifying self-concept clarity as a potential psychological resource associated with dual career adaptability among Taiwanese collegiate student-athletes. The significant indirect effect suggests that student-athletes reporting greater identity conflict also tended to report lower self-concept clarity, which was subsequently associated with lower dual career adaptability. This finding extends the application of COR theory by highlighting the possible relevance of a coherent and stable sense of self when athletes manage competing academic and athletic roles. Its contextual contribution is particularly relevant in Taiwan, where limited professional sporting opportunities mean that university frequently coincides with the peak or conclusion of an athlete’s competitive career (3). Although the findings do not establish a causal mediation process and the model explained only a limited proportion of variance in adaptability, they provide context-specific evidence that self-concept clarity warrants greater attention in cross-cultural dual career research and in the development of counselling support for Taiwanese student-athletes.

While our empirical findings reflect prospective level-associations rather than intervention-driven changes, they offer tentative proposals for university athletic support systems and student-athlete counselling in Taiwan. Previous research suggests that role models can provide narrative resources through which young athletes explore alternative identities and imagine possible future selves (33). Counselling practitioners and academic advisors might consider exploring self-concept clarity as a modest internal psychological resource when supporting student-athletes facing role strain. Institutional strategies, such as identity-mapping workshops, career-reflection sessions, or coach education, should be regarded as tentative proposals requiring rigorous pre-intervention, post-intervention, and follow-up evaluation in future research before claiming intervention effectiveness. This coordinated approach is consistent with evidence from other dual career contexts suggesting that supportive environments may facilitate the development of dual career competencies and a more sustainable balance between sport and education (34).

Furthermore, universities could consider establishing career transition programmes for student-athletes approaching graduation or athletic retirement. Possible activities could include career-interest and transferable-skills assessments, résumé preparation, job-shadowing or internship opportunities, alumni mentoring, and individualised transition planning based on structured career counselling frameworks (35). These programmes may also incorporate psychoeducational sessions on goal conflict, coping resources, and stress management. A structured intervention evaluated by Sallen, Hemming, and Richartz (36), for example, was associated with small-to-moderate improvements in stress-related knowledge, self-efficacy, and chronic stress outcomes among elite student-athletes.

### 4.1 Study limitations & future directions

This study has several limitations that should be addressed in future research. First, in the model predicting dual career adaptability, the explained variance (R²) was only about 4%. Although the indirect mechanism is significant, the low explanatory power reflects that dual career adaptability is a concept influenced by extremely multidimensional factors. Moreover, the completely standardized indirect effect was −.054, indicating that the practical magnitude of the indirect association was modest. Self-concept clarity may represent one modest psychological factor among multiple personal and contextual influences on student-athletes’ adaptation. As pointed out by Sum, Tsai, Ha, Cheng, Wang and Li (4), the socio-ecological system (e.g., family support, coaches’ attitudes, national sports policies) has a profound impact on the dual career development of elite athletes. Future research should incorporate more external social support variables, such as the students’ sense of connectedness. Additional factors may include academic support, family expectations, institutional dual career policies, training load, injury and scholarship status, and perceived professional career opportunities.

Methodological limitations must also be acknowledged when interpreting the current findings. First, regarding measurement properties, although the Self-Concept Clarity and Identity Conflict scales demonstrated good internal consistency, composite reliability (CR ≥ 0.94), and convergent validity (AVE≥.59), the elevated RMSEA values suggest that fit across all indices was not uniformly optimal. In particular, minor items such as RI4 (*λ*=.35) and RI6 (*λ*=.27) in Identity Conflict, as well as DA26 (*λ*=.30) in Dual Career Adaptability, displayed lower factor loadings. Hence, Self-Concept Clarity should be considered as a preliminary and modest internal psychological resource rather than a primary or sole mechanism explaining dual career adjustment. Future research should refine these measures and evaluate measurement invariance across diverse competitive levels and sport types.

In addition, the primary mediation hypothesis was evaluated using observed-variable path analysis via the PROCESS macro (Model 4) (23). While path models based on composite scores do not explicitly control for random measurement error—which can potentially bias indirect effect estimates (31)—methodological evaluations show that when scale reliabilities are exceptionally high (CR ≥ 0.94), observed-variable regression and latent-variable SEM yield virtually identical indirect effect estimates (32). To confirm robustness, a full latent-variable SEM re-estimation was conducted in LISREL, which confirmed that the latent indirect effect of Identity Conflict on Dual Career Adaptability via Self-Concept Clarity remained statistically significant [Bootstrap 95% CI: (−0.062,−0.008), excluding zero] (29). Nonetheless, future longitudinal studies employing multi-wave designs (T ≥ 3) and full latent structural equation modelling are encouraged to replicate these indirect pathways while rigorously controlling for baseline construct levels and measurement error.

Although the two-wave design with a four-month interval provided temporal separation between self-concept clarity and dual career adaptability, identity conflict and self-concept clarity were measured concurrently at T1. Therefore, the temporal ordering between the predictor and mediator cannot be established. Future research should adopt a minimum three-wave design in which identity conflict is measured at T1, self-concept clarity at T2, and dual career adaptability at T3, while controlling for their baseline levels. Such research could span an entire academic year or major educational and career transitions, such as the transition from high school to university or from university to employment, to examine how these relationships develop across different stages of athletes’ dual careers (2). Also, future longitudinal research could systematically examine selective attrition in two- or three-wave surveys of student-athletes by comparing the demographic, athletic, and psychological characteristics of participants who remain in the study with those who withdraw across successive waves.

In addition, although identity conflict represents a central and observable manifestation of tension between student and athlete identities, the Student-Athlete Role Interference Scale primarily captures practical incompatibilities involving time, performance, and role expectations and may not encompass deeper psychological tensions between different self-definitions. In addition, although several CFA indices indicated acceptable fit, the elevated RMSEA and *χ*²/df values suggest that the measurement model did not demonstrate uniformly satisfactory fit. The findings involving identity conflict should therefore be interpreted in accordance with this operational definition. Future research should further validate and refine the Chinese version of the scale, compare it with alternative measures of identity conflict, and examine its measurement invariance across different sports, genders, and educational settings.

Finally, the study did not test whether counselling, narrative interventions, or institutional support programs improve self-concept clarity or dual career adaptability. These practical implications should therefore be regarded as possible applications. Future experimental or quasi-experimental studies could evaluate identity-based counselling, structured career-reflection workshops, and coach training through pre-intervention, post-intervention, and follow-up assessments of self-concept clarity and dual career adaptability. Finally, because the study focused on Taiwanese collegiate student-athletes, its findings may not generalise directly to student-athletes operating within different educational, sporting, and professional systems. Cross-cultural replication is needed to determine whether the observed associations are consistent across socio-ecological contexts.

## 5 Conclusion

This study provides empirical evidence from a two-wave prospective association model with a cross-sectional first stage. The results indicate that student-athletes experiencing higher academic–athletic role interference at Time 1 report lower self-concept clarity at Time 1, which in turn is associated with lower dual career adaptability measured four months later at Time 2. We emphasize that these findings represent prospective indirect level-associations rather than temporal mediation or longitudinal change over time. Future research utilizing full three-wave longitudinal designs with baseline controls is necessary to establish temporal precedence and capture intra-individual change trajectories over time.

These findings highlight that the challenges faced by student-athletes may involve not only the presence of multiple role demands but also their ability to maintain a clear understanding of who they are and how they may move forward across academic, athletic, and future career pathways. In the Taiwanese context, where professional sport opportunities remain limited, and university often represents a critical transition point, supporting student-athletes’ self-concept clarity may be a relevant consideration for educators, coaches, and counselling practitioners. Efforts to strengthen this psychological resource may help student-athletes manage role conflict during their development, although this possibility requires further longitudinal and intervention-based research. Overall, this study contributes to dual career research by identifying self-concept clarity as a potential psychological pathway linking identity conflict and adaptive outcomes among collegiate student-athletes.

## Data Availability

The raw data supporting the conclusions of this article will be made available by the authors, without undue reservation.
