# Supplementary material for: A study on identity conflict, self-concept clarity, and dual career adaptability among collegiate athletes
Source: Front Sports Act Living. 2026 Sep 14;8:1877707. doi: 10.3389/fspor.2026.1877707 (PMC13616655; doi:10.3389/fspor.2026.1877707)
Supplement: Supplementary file 1 [file Table1.docx]

Supplementary Material

## Tables

Table 1

Results of variable distribution and correlation analysis.

| **Variables** | **1** | **2** | **3** | **4** | **5** | **6** |
| --- | --- | --- | --- | --- | --- | --- |
| 1. gender | -- |  |  |  |  |  |
| 2. age | -.03 | -- |  |  |  |  |
| 3. years of training | -.20^**^ | .46^**^ | -- |  |  |  |
| 4. identity conflict | .04 | -.01 | .09 | -- |  |  |
| 5. self-concept clarity | .07 | .08 | .01 | -.35^**^ | -- |  |
| 6. dual career adaptability | .11^*^ | .07 | -.02 | -.02 | .16^**^ | - |
| Mean | - | 20.24 | 10.52 | 4.00 | 3.22 | 3.92 |
| Standard Deviation | - | 1.75 | 3.20 | .96 | .93 | .59 |

Note: N = 414. Gender was coded as a dummy variable. **p* < .05 ***p* <.01 ****p* < .001

Table 2

*Integrated Regression and Effect Decomposition Results for the Prospective Indirect Model (N = 414)*

| Model & Path / Parameter | | Unstd. B | | *SE* | | *t* */ Boot SE* | | p-value | | 95% CI [LL, UL] | | Std. *β* | |
| --- | --- | --- | --- | --- | --- | --- | --- | --- | --- | --- | --- | --- | --- |
| Mediator Model: Self-Concept Clarity(T1), R^2^ = .133，F_（4, 409）_= 15.68, *p* <.001 | | | | | | | | | | | |  | |
| Constant | | 3.685 | | .543 | | 6.785 | | < .001 | | [2.618, 4.753] | | — | |
| Gender  (0=Male,1= Female) | | .167 | | .088 | | 1.894 | | .059 | | [-0.006, 0.341] | | .088 | |
| Age | | .037 | | .028 | | 1.327 | | .185 | | [-0.018, 0.091] | | .069 | |
| Years of Training | | .005 | | .016 | | 0.292 | | .770 | | [-0.026, 0.035] | | .015 | |
| Identity Conflict (T1) | | -.339 | | .045 | | -7.558 | | < .001 | | [-0.427, -0.251] | | -.350 | |
| Outcome Model: Dual Career Adaptability (T2), R^2^ = .040，F_（5, 408）_= 3.42 , *p* = .005 | | | | | | | | | | | |  | |
| Constant | | 2.980 | | .383 | | 7.790 | | < .001 | | [2.228, 3.731] | | — | |
| Gender | | .107 | | .059 | | 1.808 | | .071 | | [-0.009, 0.223] | | .089 | |
| Age | | .028 | | .019 | | 1.521 | | .129 | | [-0.008, 0.065] | | .082 | |
| Years of Training | | -.008 | | .010 | | -0.754 | | .451 | | [-0.028, 0.013] | | -.042 | |
| Identity Conflict (T1) [Direct Effect, c'] | | .02 | | .032 | | 0.627 | | .531 | | [-0.043, 0.083] | | .032 | |
| Self-Concept Clarity (T1) [Mediator, b] | | .098 | | .033 | | 2.973 | | .003 | | [0.033, 0.163] | | .154 | |
| Effect Decomposition Summary (Identity Conflict T1 → Dual Career Adaptability T2) (5,000 Bootstrap) | | | | | | | | | | | | | |
| Total Effect (c) | -.013 | | .032 | | -0.414 | | .679 | | [-0.076, 0.050] | | -.022 | |  |
| Direct Effect (c') | .020 | | .032 | | 0.627 | | .531 | | [-0.043, 0.083] | | .032 | |  |
| Indirect Effect (a × b) via SCC | -.033 | | .015 | | — | | .012 | | [-0.069, -0.007] | | -.054 | |  |
| *Note.* N = 414. Gender was dummy-coded (0 = Male, 1 = Female). Bootstrap resamples = 5,000. *For the indirect effect, Boot SE and 95% Bias-Corrected Bootstrap Confidence Intervals are reported. Completely standardized indirect effect β = -.054 (Boot SE = .024, 95% Boot CI [-.112, -.011]). The direct effect (c' = +0.020) and indirect effect (a × b = -0.033) operate in opposite directions, resulting in a near-zero total association (c = -0.013, p = .679; bivariate r = -.02), which reflects inconsistent mediation (or statistical suppression) (Cheung & Lau, 2008; Kline, 2016; MacKinnon et al., 2000). **p* < .05, ***p* < .01, ****p* < .001. | | | | | | | | | | | |  | |
